# Hydrogenation-Facilitated Spontaneous N-O Cleavage Mechanism for Effectively Boosting Nitrate Reduction Reaction on Fe_2_B_2_ MBene

**DOI:** 10.3390/molecules30081778

**Published:** 2025-04-15

**Authors:** Yuexuan He, Zhiwen Chen, Qing Jiang

**Affiliations:** Key Laboratory of Automobile Materials, Ministry of Education, School of Materials Science and Engineering, Jilin University, Changchun 130022, China; yxhe21@mails.jlu.edu.cn

**Keywords:** MBene catalyst, NO_3_^−^ reduction reaction, density functional theory, electrochemical catalysis, reaction mechanism

## Abstract

The electrochemical reduction of toxic nitrate wastewater to green fuel ammonia under mild conditions has become a goal that researchers have relentlessly pursued. Existing designed electrocatalysts can effectively promote the nitrate reduction reaction (NO_3_RR), but the study of the catalytic mechanism is not extensive enough, resulting in no breakthroughs in performance. In this study, a novel mechanism of hydrogenation-facilitated spontaneous N-O cleavage was explored based on density functional theory calculations. Furthermore, the *E*_ad−*OH_ (adsorption energy of the adsorbed *OH) was used as a key descriptor for predicting the occurrence of spontaneous N-O bond cleavage. We found that *E*_ad−*OH_ < −0.20 eV results into spontaneous N-O bond cleavage. However, excessively strong adsorption of OH* hinders the formation of water. To address this challenge, we designed the eligible Fe_2_B_2_ MBene, which shows excellent catalytic activity with an ultra-low limiting potential for NO_3_RR of −0.22  V under this novel reaction mechanism. Additionally, electron-deficient Fe active sites could inhibit competing hydrogen evolution reactions (HERs), which provides high selectivity. This work may offer valuable insights for the rational design of advanced electrocatalysts with enhanced performance.

## 1. Introduction

Nitrate (NO_3_^−^) has become a common, environmentally harmful pollutant, especially abundant in domestic and industrial wastewater [1]. Consequently, removing NO_3_^−^ pollutant from water sources has become a worldwide and urgent problem [2,3,4]. To overcome the pollution problem, significant efforts have been made to developing sustainable technologies for converting pollutants into viable energy sources [5,6]. Specifically, the conversion of NO_3_^−^ pollution to harmless value-added ammonia (NH_3_) by electrochemical reduction has received much attention [7,8,9,10], which protects the environment while gaining valuable energy. NH_3_ serves as a crucial chemical feedstock for fertilizer production while simultaneously emerging as a promising candidate for clean energy storage and sustainable fuel applications [11,12,13]. However, the industrial production of NH_3_ remains predominantly reliant on the energy-intensive Haber–Bosch (H-B) process, which operates under extreme reaction conditions (350–500 °C and 150–350 atm), accounting for approximately 1–2% of global energy consumption and contributing significantly to greenhouse gas emissions [14,15,16,17,18]. The electrocatalytic synthesis of NH_3_ by NO_3_^−^ reduction reaction (NO_3_RR) at ambient conditions is considered a promising alternative to the H-B method [19]. However, electrocatalytic NO_3_RR is a complex and difficult process involving multiple electron transfers, while also inevitably competing with hydrogen evolution reactions (HERs). Thus, there is an urgent need to find efficient and highly selective catalysts for NO_3_RR.

The design of catalysts under traditional reaction mechanisms is guided by the Sabatier principle [20,21], while the inherent linear scaling relationships between adsorption strengths of multiple similar intermediates impose fundamental limitations on catalytic activity, creating significant challenges in performance optimization. Normally, the first hydrogenation (*NO_3_ → *NO_3_H) step usually has a large change in Gibbs reaction free energy (Δ*G*) [22,23], indirectly suggesting that the insufficient intermediate interactions may exacerbate the difficulty of activation [24,25,26]. Undoubtedly, the generally accepted reaction mechanism makes it difficult to break this relationship of a significant increase in energy for hydrogenation without deoxygenation, clearly implying that an innovative understanding of the NO_3_RR reaction mechanism is necessary.

Surprisingly, the intermediates are found to be lower in energy after N-O bond cleavage than before [27]. However, this non-spontaneous N-O bond cleavage needs to overcome a large energy barrier, making the reaction difficult. Inspired by these thoughts, a brand-new reaction mechanism of hydrogenation-facilitated spontaneous N-O cleavage is proposed for NO_3_RR, which circumvents the inherent limitations of conventional reaction mechanisms, and is expected to accelerate hydrogenation and N-O cleavage simultaneously, resulting in better catalytic performance.

Fortunately, we detected hydrogenation-facilitated spontaneous N-O cleavage on traditional metal catalysts (such as Fe, Co, Ni and Cu) when *E*_ad-*OH_ < −0.20 eV. Fe has shown the most negative *E*_ad−*OH_ of the above metals; however, the catalytic performance of Fe for NO_3_RR is not satisfactory [28]. The essential reason is that the strong adsorption of Fe is unfavorable for intermediates releasing, which can be regulated efficiently by modulating the electronic structure and coordinated environment. Notably, B could utilize empty orbitals to accept electrons from Fe-3*d* orbitals inducing local orbital electron-deficient Fe sites, which help regulate adsorption. These moderate electron-deficiency Fe sites without excessive electron loss do not hinder the “acceptance–donation” behavior between Fe-3*d* orbitals and NO_3_^−^-π* orbitals, implying that Fe_2_B_2_ still has the potential for high NO_3_RR catalytic activity. In addition, Fe sites with positive charge have poor binding strength with protons, which is able to improve the NH_3_ synthesis selectivity.

With these thoughts in mind, a novel transition metal boride (MBene) catalyst, Fe_2_B_2_, is designed in this work according to the proposed mechanism. We investigate the NO_3_^−^ electroreduction catalytic performance of Fe_2_B_2_ through density functional theory (DFT) calculations. Abundant Fe active sites on the surface enables hydrogenation-facilitated spontaneous N-O cleavage. Also, the electron-deficient Fe atoms result in the inhibition of the competitive HER. Our results demonstrate that Fe_2_B_2_ shows exceptional catalytic performance for NO_3_RR with an ultra-low potential of −0.22 V under the new mechanism with superior selectivity.

## 2. Results and Discussion

### 2.1. Hydrogenation-Facilitated Spontaneous N-O Cleavage Mechanism

**Figure 1:**
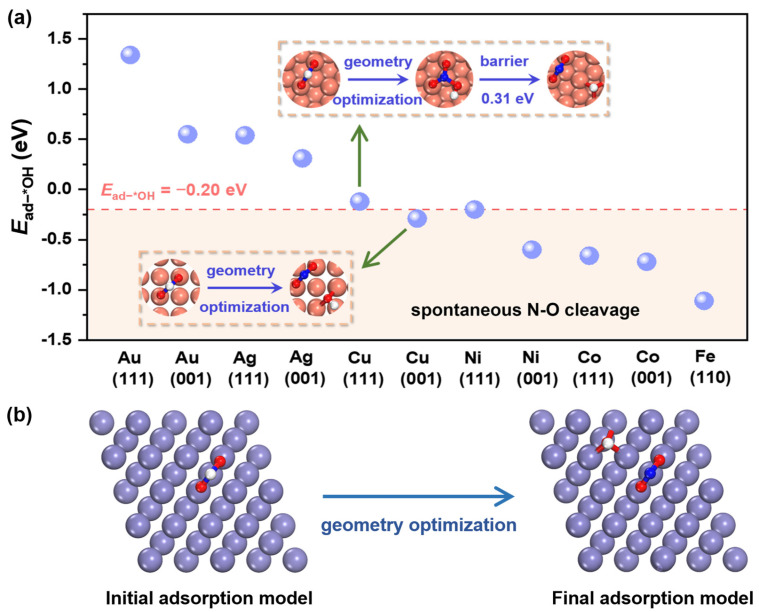
(**a**) The adsorption energies of *OH (*E*_ad−*OH_) on several TM surfaces. (**b**) The NO_3_H adsorption configurations before (**left**) and after (**right**) geometry optimization on Fe (110).

To explore hydrogenation-facilitated spontaneous N-O cleavage mechanism, the first hydrogenation of *NO_3_ (*NO_3_H) on several transition metal (TM)-stabilized surfaces are calculated, including the (110) facet of Fe, as well as the (111) and (001) facets of Co, Ni, Cu, Ag and Au (Figure S1, Supplementary Materials). Simultaneously, the adsorption energies of *OH (*E*_ad−*OH_) on corresponding surfaces are calculated, as illustrated in Figure 1a. Selecting *E*_ad−*OH_ as an effective descriptor, a tendency for spontaneous N-O cleavage is established where there is a threshold. As a groundbreaking finding, the N-O bond of *NO_3_H intermediate cleaves spontaneously when the value of *E*_ad−*OH_ is negative than −0.20 eV. Cu-based catalysts are one of the most promising catalysts available for NO_3_RR [29], whose first hydrogenation step is the potential-determining step (PDS) of the whole pathway, based on previous studies [30]. The Δ*G* of its first hydrogenation step is calculated as 0.48 eV with the optimized structure pictured in Figure 1a, which is still hard to overcome. In addition, N-O bond-breaking in a *NO_3_H intermediate on Cu catalyst is also kinetically difficult, as evidenced by calculating the energy barrier through transition state (TS) searching (Figure S2). Hence, even though Cu catalyst is one of the high-activity catalysts, there is still space to improve the catalytic performance for NO_3_RR.

Fe has the most negative *E*_ad−*OH_, as shown in Figure 1a, giving rise to the prediction that it would enable the easiest spontaneous N-O cleavage, whose structural optimization process is illustrated in Figure 1b. However, too strong an adsorption is not conducive to the subsequent transformation–desorption of intermediate substances [31,32]. To address this issue, Fe_2_B_2_ MBene has been proposed. The *d*-band center (Ɛ*_d_*) of Fe_2_B_2_ and Fe calculated are −2.24 and −2.14 eV, respectively (Figure S3). Compared with Fe, the Ɛ*_d_* of Fe_2_B_2_ is farther from Fermi level, resulting in a tendency for weaker adsorption [33,34]. Notably, *E*_ad−*OH_ of Fe_2_B_2_ calculated is −0.47 eV, which meets the criterion of less than −0.20 eV for the hydrogenation-facilitated spontaneous N-O cleavage mechanism.

### 2.2. Structures and Stability of Fe_2_B_2_

**Figure 2:**
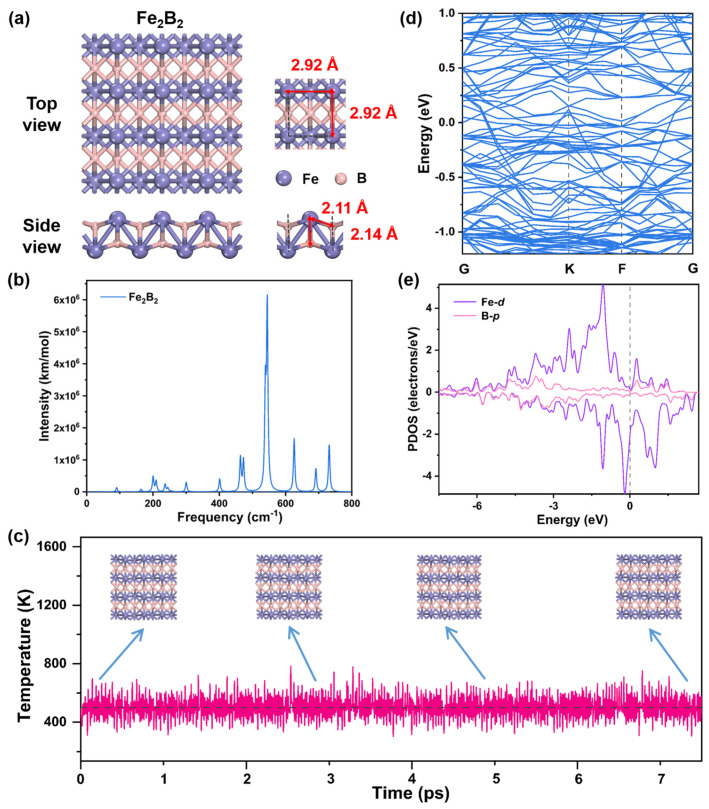
(**a**) The geometric structures of the Fe_2_B_2_ surface with the corresponding distance between atoms. The purple and pink balls represent Fe and B atoms, respectively. (**b**) The frequency distribution of geometrically optimized Fe_2_B_2_. (**c**) Four structural fragments from MD simulation during a total time of 7.5 ps. (**d**) The band structure of Fe_2_B_2_. (**e**) The PDOS of Fe and B atoms of the Fe_2_B_2_ surface. The Fermi level is set at an energy of zero, as indicated by the gray dotted line.

The optimized geometric structure of pristine 2D Fe_2_B_2_ is illustrated in Figure 2a, where Fe active sites are fully exposed on the surface. The crystal structure of layered Fe_2_B_2_ is an accordion-like configuration composed of layers of B atoms inserted into the subsurface of Fe-based frameworks. The dynamical stability of Fe_2_B_2_ was assessed by frequency calculations. Figure 2b presents the frequency dispersion analysis of the fully optimized Fe_2_B_2_ structure, which exhibits no significant imaginary frequencies, confirming its kinetic stability. The thermodynamic stability of Fe_2_B_2_ is also evaluated through the molecular dynamics (MD) simulation with a constant temperature of *T* = 500 K (Figure 2c). Four snapshots of Fe_2_B_2_ at the time of 0.3, 2.9, 5.0, and 7.2 ps, respectively, are almost the same, providing strong evidence for the high thermodynamic stability of the system. The exceptional dynamical and thermodynamic stability of Fe_2_B_2_ ensures sustained catalytic performance and long-term durability in NO_3_RR applications. Simultaneously, MBenes are experimentally considered to have good stability in acid-base solutions. Fe_2_B_2_, as one of the MBenes, is expected to likewise have this property [35]. Moreover, the interaction between Fe and B atoms is verified by the charge density difference (Figure S4), where the pronounced charge redistributions indicate strong chemical interactions. From Hirshfeld charge analysis, the electron transfers from Fe to B in the Fe_2_B_2_ system and the surface Fe atom has a positive charge of +0.17 e. The direction of electron transfer is consistent with the Pauling electronegativity values for Fe (1.83) and B (2.04) [36].

To understand the electronic properties of Fe_2_B_2_, we calculated the band structure as shown in Figure 2d. Obviously, there is no band gap near the Fermi level and some bands across the Fermi level, which means there are metallic conductor characteristics. Additionally, to further investigate the electron structure of Fe_2_B_2_, the partial density of states (PDOS) is calculated to evaluate the interaction between B and Fe atoms (Figure 2e). There are significant hybridizations between Fe-3*d* and adjacent B-2*p* orbitals revealed by the strong overlaps. The strong interaction and stable chemical bond between Fe and B atoms are confirmed by the PDOS projected on the *d* orbitals and the *p* orbitals.

### 2.3. NO_3_^−^ Adsorption and Activation

**Figure 3:**
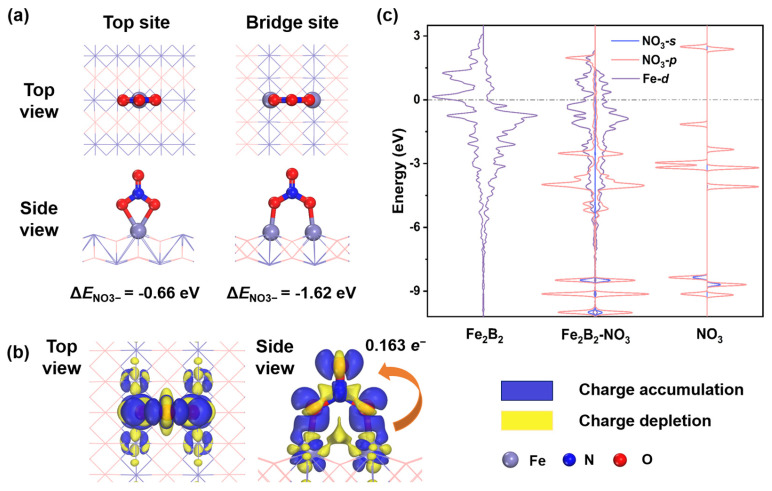
(**a**) The top and side views for NO_3_^−^ adsorbed at different adsorption sites on Fe_2_B_2_ surface and the corresponding adsorption energy values. (**b**) The electron density difference of NO_3_^−^ adsorbed at the bridge site of Fe_2_B_2_ surface, where blue and yellow regions denote electron accumulation and depletion, respectively. (**c**) The PDOS of Fe atoms on Fe_2_B_2_ surface, NO_3_^−^ adsorbed at the bridge site and an isolated NO_3_^−^ molecule.

The adsorption of NO_3_^−^ on Fe_2_B_2_ as the initial step is one of the necessary conditions for ensuring the smooth progress of the NO_3_RR. Given the structure of Fe_2_B_2_, two adsorption configurations are considered: NO_3_^−^ on the top site and on the bridge site, as illustrated in Figure 3a. The calculated adsorption energy (*E*_ad_) values of NO_3_^−^ on the top and bridge sites are −0.66 and −1.62 eV, respectively. Thus, the bridge site NO_3_^−^ is the optimal adsorption configuration, which will be focused on in the following section. In view of the Hirshfeld charge analysis, 0.163 e^−^ transfers into the NO_3_^−^ molecule. The charge density difference between Fe_2_B_2_ and the adsorbed NO_3_^−^ in Figure 3b obviously shows electron transfer. The charge acceptance and donation take place on both Fe atoms and the NO_3_^−^ molecule, which are responsible for the activation of NO_3_^−^. Additionally, unpaired electrons in Fe-3*d* orbitals decrease after adsorption, leading to a significant reduction in the magnetic moment of Fe atom from 1.56 μB to 0.45 μB. The main reason for the reduction in total magnetic moment is that the empty *d* orbitals in the Fe atoms could receive the lone-pair electrons of NO_3_^−^, which means that the charge transfer between Fe and NO_3_^−^ induces a change in the spin magnetic moment.

In addition, to gain fundamental insights into the bonding characteristics during NO_3_^−^ activation, we conducted a comparative analysis of the interaction between NO_3_^−^ and Fe_2_B_2_ using the projected density of states (PDOS) before and after adsorption (Figure 3c). Before NO_3_^−^ adsorption, Fe-3*d* states possess strong peaks around Fermi level, which facilitates efficient electron transfer during the NO_3_^−^ activation. After NO_3_^−^ adsorption, significant orbital overlap between O-2*s* and -2*p* orbitals and Fe-3*d* orbitals emerges, demonstrating that the hybridizing occurs. The Fe-3*d* orbitals accept the lone-pair electrons from the NO_3_^−^ molecule to form stable bonding states. These results reveal that the NO_3_^−^ molecule is effectively activated and active for subsequent protonation.

### 2.4. NO_3_RR Performance of Fe_2_B_2_

**Figure 4:**
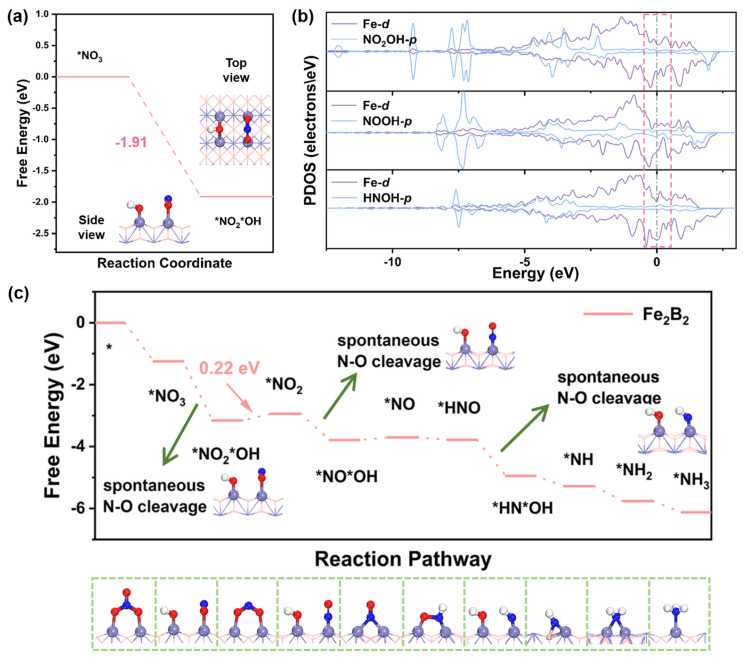
(**a**) The reaction free energy changes of *NO_3_ to *NO_2_*OH on Fe_2_B_2_ and the corresponding structures of decomposed *NO_2_*OH adsorbed on the Fe_2_B_2_ surface. (**b**) The PDOS of Fe atoms and *NO_2_*OH, *NO*OH and *HN*OH on the Fe_2_B_2_ surface. (**c**) The Gibbs free energy change diagram for NO_3_RR and the corresponding adsorption configuration of intermediates on the Fe_2_B_2_ surface (* represents the active site).

The potential for nitrate protonation must be assessed before examining the specific reaction pathway. We find that Fe_2_B_2_ satisfies the criterion (*E*_ad−*OH_ < −0.20 eV) to achieve N-O cleavage. There still are numerous bare active sites on the Fe_2_B_2_ surface after capturing the NO_3_^−^ molecule. With the aid of these active sites, *NO_3_H is decomposed into *NO_2_ and *OH fragments, attributed to a cleavage effect. The reaction Gibbs free energy of *NO_2_*OH formation (ΔG_*NO2*OH_) calculated is −1.91 eV, indicating that the N-O bond cleaves spontaneously, while the adsorption configuration is also considered in Figure 4a. We further calculate the PDOS of decomposed *NO_2_*OH on Fe_2_B_2_ surface. The PDOS in Figure 4b shows the significant orbital hybridization between Fe_2_B_2_ and *NO_2_*OH where the remarkable overlap of *p*-*d* orbitals near the Fermi level is present. This mitigates the inherent intrinsic linkage of the intermediate and hinders the energy rise. Analogously, the cleavage is also realized in *NO*OH and *HN*OH on Fe_2_B_2_ (Figure S5 and Figure 4b). The corresponding structures before and after the optimization of each hydrogenation-facilitated N-O cleavage step are provided in Figure S6.

On these bases, a brand-new reaction mechanism called spontaneous N-O cleavage mechanism for NO_3_RR on Fe_2_B_2_ is put forward, as exhibited in Figure S7. For the electron-deficient surface of Fe_2_B_2_, the NO_3_^−^ molecule is first adsorbed at the bridge site with ∆G_*NO3−_ = −1.25 eV and then the follow protonation steps based on the new mechanism (Figure 4c). After the first hydrogenation step, protonation initiates the decomposition of intermediate *NO_3_H into *NO_2_ and *OH fragments. The ∆*G* value for the formation of *NO_2_*OH is −1.91 eV. Subsequently, the H proton tends to attack *OH to form the first H_2_O molecule and release from Fe_2_B_2_ surface, which is the PDS with the maximal ∆*G* value of 0.22 eV and *U*_L_ = −0.22 V vs. RHE. Likewise, the *NO_2_H separates into *NO and *OH with ∆*G*  =  −0.85 eV in the third hydrogenation process. After that, the second H_2_O molecule also continually releases with a ∆*G* value of −0.09 eV. Next, the free energy changes for both pathways of *NO hydrogenation (*NO → *HNO and *NO → *NOH) are calculated with the detailed results provided in Figure S8. The formation of *NOH is undesirable with ΔG_*NOH_ = 0.79 eV, which is much higher than ΔG_*HNO_ (−0.08 eV), suggesting that the *HNO pathway is better. Hence, *HNO hydrogenates to decomposed *NH and *OH fragments (ΔG_*HN*OH_ = −1.17 eV). In subsequent reaction steps, the protons continue to drive the transformation of remaining intermediates, ultimately yielding the third H_2_O molecule and the desired NH_3_ product. The ∆*G* value for releasing H_2_O is −0.33 eV. And ∆*G* values for *NH_2_ and *NH_3_ formations are −0.48 and −0.36 eV, respectively. Finally, we performed comparative calculations of NO_3_RR performance between Fe(110) and Fe_2_B_2_ (Figure S9). The results show that the maximal Δ*G* of Fe is 0.91 eV, which is much higher than that of Fe_2_B_2_ (0.22 eV). Furthermore, we also considered the implicit solvation effect and the calculation shows that the maximal ∆*G* value for PDS of Fe_2_B_2_ is 0.31 eV, confirming that Fe_2_B_2_ still maintains excellent NO_3_RR catalytic activity under this condition (Figure S10). So far, the ability of this reaction mechanism to effectively lower the energy changes has been almost verified. Encouragingly, compared with traditional reaction mechanism, the novel mechanism of the catalysis ensures the stability of adsorption and promotes the activation step through spontaneous N-O cleavage, thereby strongly validating our initial hypothesis.

### 2.5. Selectivity Toward NH_3_ Synthesis

Furthermore, owing to the presence of competitive reaction of HER, selectivity is the focus of NO_3_RR catalysts in addition to high catalytic activity. An optimal NO_3_RR electrocatalyst must mitigate detrimental H species from poisoning and inhibit the competing HER during the NH_3_ synthesis process. The initial hydrogen adsorption step is a prerequisite and fundamental thermodynamic requirement for the entire HER process. Here, the calculated H^+^ adsorption free energy (∆E_*H_) on Fe sites is −0.27 eV, which is more positive than ∆E_*NO3_ (Figure S11), suggesting that NO_3_^−^ rather than H^+^ tends to adsorb on the active sites. Obviously, the electron-deficient Fe sites contribute to the superior selectivity. Additionally, we also calculate the adsorption of H^+^ in the presence of an applied corresponding limiting potential to comprehensively evaluate the adsorption selectivity under the corresponding *U*_L_ of NO_3_RR (Figure S11). The negative value of −0.49 eV shows the adsorption of NO_3_^−^ remains much stronger than that of H^+^. These results imply that the active sites still preferentially adsorb NO_3_− at the operating potential, indicating that Fe_2_B_2_ still has an excellent selectivity towards NO_3_RR.

## 3. Computational Methods

In this study, all first-principles calculations were performed using spin-polarization density functional theory (DFT) and implemented in the Dmol^3^ code in Material Studio [37,38]. The exchange–correlation effect is treated by the generalized gradient approximation (GGA) with Perdew–Burke–Ernzerhof (PBE) functional [39,40]. The Grimme method for DFT-D correction is applied to describe the van der Waals forces [41]. The DFT semi-core pseudopotential (DSPP) method is implemented for the treatment of core electrons with the basis set of double numerical plus polarization (DNP) set as 4.4 [38,42]. The convergence criteria is set to 1.0 × 10^−5^ hartree (Ha) for the energy change, 2.0 × 10^−3^ Ha Å^−1^ for the gradient and 5.0 × 10^−3^ Å for the displacement, respectively. A smearing value of 0.005 Ha is chosen to speed up convergence. All atoms are fully relaxed during the calculations. The 3 × 3 supercell of 2D Fe_2_B_2_ (001) with 4 layers of atoms is built and the vacuum gap is 15 Å to avoid the interaction between periodic structures in *z*-direction. The *k*-point grid is set as 4 × 4 × 1 in Brillouin zone. Before geometrical optimization, the initial spin of Fe is set to 4. The atomic charges are calculated by Hirshfeld analysis to further study the nature of charge transfer [43,44]. To investigate the N-O bond cleavage, linear synchronous transit/quadratic synchronous transit (LST/QST) methods of the transition state (TS) are used [45].

In this work, the adsorption energy (*E*_ad−*M_, M denotes the corresponding species) is defined as

*E*_ad−*M_ = E_*M_ − E_*_ − *E*_M_

where E_*M_ represents the total energy of the catalyst and adsorbed material, E_*_ is the energy of the isolated catalyst and *E*_M_ is the energy of the corresponding adsorbate molecule in the independent gas state.

The electrochemical NO_3_RR process involved net transfer of 9 protons and 8 electrons (NO_3_^−^ + 9H^+^ + 8e^−^ → NH_3_ + 3H_2_O) and is calculated based on the computational hydrogen electrode (CHE) model proposed by Nørskov et al. [46,47]. The free energy of a proton–electron pair at the chemical potential of 0 V vs. RHE under standard reaction conditions (pH = 0, 298.15 K, 1 atm) is equivalent to 1/2 H_2_(g) [48,49]. According to this theory, the Gibbs reaction free energy change (Δ*G*) is determined by

Δ*G* = Δ*E* + Δ*ZPE* − *T*Δ*S*

where Δ*E*, Δ*ZPE*, *T* and Δ*S* are the change in electronic reaction energy, the zero-point energy (ZPE), the temperature (298.15 K) and the entropy difference between products and reactants, respectively. In addition, the limiting potential (*U*_L_) of each step is defined as

*U*_L_ = −Δ*G*_max_/*e*

where Δ*G*_max_ denotes the Gibbs free energy change at the highest elementary step for the pathway during the NO_3_^−^ reduction process, which means the potential-determining step. The entropy values for gas-phase molecules are obtained from the standard values of thermodynamics [50].

## 4. Conclusions

In conclusion, we used DFT calculations to synthetically investigate the potential of Fe_2_B_2_ as an efficient and selective electrocatalyst for NO_3_RR. Using Fe_2_B_2_ as a prototype, guided by cleavage idea, we have successfully proposed a brand-new mechanism for NO_3_RR, namely a spontaneous N-O cleavage mechanism. As found, Fe_2_B_2_ exhibits remarkable NO_3_RR performance with an ultra-low limiting potential of −0.22 V vs. RHE under the new mechanism. In addition, electron-deficient Fe active sites also exhibit outstanding NO_3_RR selectivity and significantly inhibit the competing HER, even at the applied potential of NO_3_RR. Thus, Fe_2_B_2_ emerges as a promising candidate electrocatalyst for NO_3_RR due to its outstanding catalytic activity and superior selectivity. Collectively, our work establishes a novel reaction mechanism and provides valuable guidance for future catalyst design.

## Data Availability

Data are contained within the article.

## Supplementary Materials

The following supporting information can be downloaded at: https://www.mdpi.com/article/10.3390/molecules30081778/s1, Figure S1: The geometry optimized *NO_3_H (*NO_2_*OH) adsorption configurations on the surface of transition metals (TM). Figure S2: Dissociation process of *NO_3_H to *NO_2_*OH on Cu (111). Figure S3: The partial density of states (PDOS) plots of Fe_2_B_2_ and Fe (110). The *d*-band centers are also highlighted in the PDOS curves. Figure S4: The atomic electron density maps of Fe_2_B_2_ surface. The blue and yellow isosurfaces represent electron density accumulation and depletion, respectively. Figure S5: (a) The reaction free energy changes of *NO_2_ to *NO*OH and the corresponding structure on Fe_2_B_2_ surface. (b) The reaction free energy changes of *HNO to *HN*OH and the corresponding structure on Fe_2_B_2_ surface. Figure S6: The corresponding adsorption configurations of *NO_3_H (*NO_2_*OH), *NO_2_H (*NO*OH) and *HNOH (*HN*OH) before (left) and after (right) geometry optimization on Fe_2_B_2_ surface. Figure S7: The brand-new reaction mechanism of intermediate separation strategy. Figure S8: Protonation of *NO on Fe_2_B_2_, including two competitive pathways of *NOH and *HNO. Figure S9: The Gibbs free energy change diagram for NO_3_RR on Fe_2_B_2_ surface and Fe(110). Figure S10: The Gibbs free energy change diagram for NO_3_RR on Fe_2_B_2_ surface incorporating the implicit solvation effect. Figure S11: The calculated the adsorption energy of *NO_3_ and *H on Fe_2_B_2_ surface.
