# Supplementary material for: Hydrogenation-Facilitated Spontaneous N-O Cleavage Mechanism for Effectively Boosting Nitrate Reduction Reaction on Fe2B2 MBene
Source: Molecules. 2025 Apr 15;30(8):1778. doi: 10.3390/molecules30081778 (PMC12029549; doi:10.3390/molecules30081778)
Supplement: Supplementary file 1 [file molecules-30-01778-s001.zip › molecules-3566790-supplementary.pdf]

Supporting Information

Hydrogenation facilitated spontaneous N-O cleavage mecha-nism for effectively boosting nitrate reduction reaction on Fe_2B_2 MBene

Yuexuan He, Zhiwen Chen*, Qing Jiang*

Key Laboratory of Automobile Materials, Ministry of Education, and School of Materials Science and Engineering, Jilin University, Changchun 130022, China

*Corresponding author. Zhiwen Chen (zwchen@jlu.edu.cn) and Qing Jiang (jiangq@jlu.edu.cn)

* Correspondence and requests for materials should be addressed to Z. C. and Q. J. (e-mail: zwchen@jlu.edu.cn, jiangq@jlu.edu.cn).

Supplementary Figures.

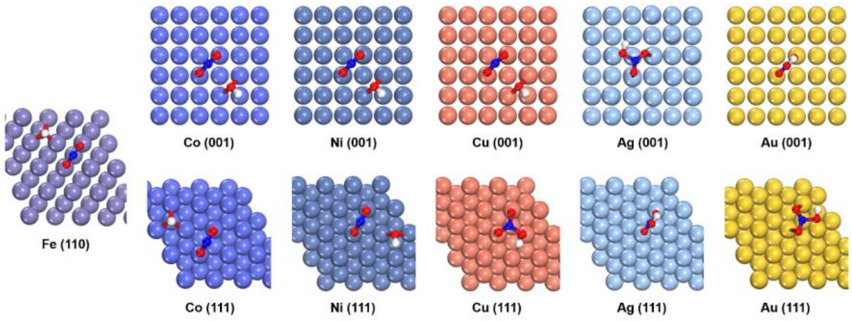

Figure S1. The geometry optimized $^*NO_3H$ ($^*NO_2{}^*OH$) adsorption configurations on the surface of transition metals (TM).

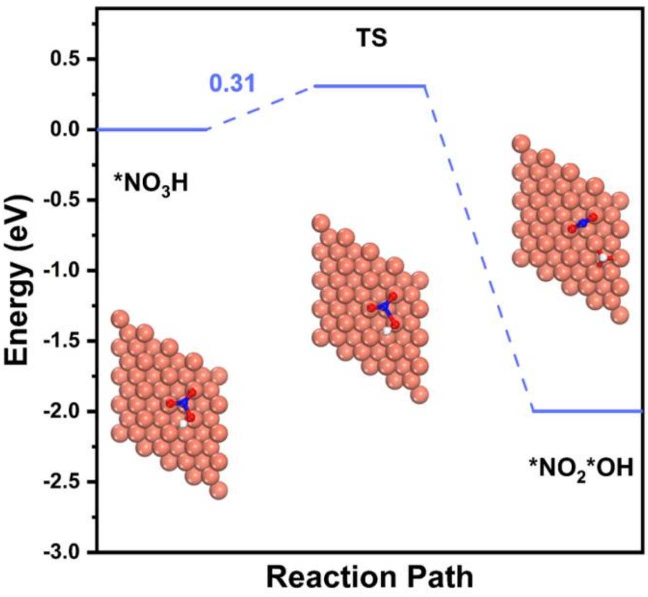

Figure S2. Dissociation process of $*NO_3H$ to $*NO_2*OH$ on Cu (111).

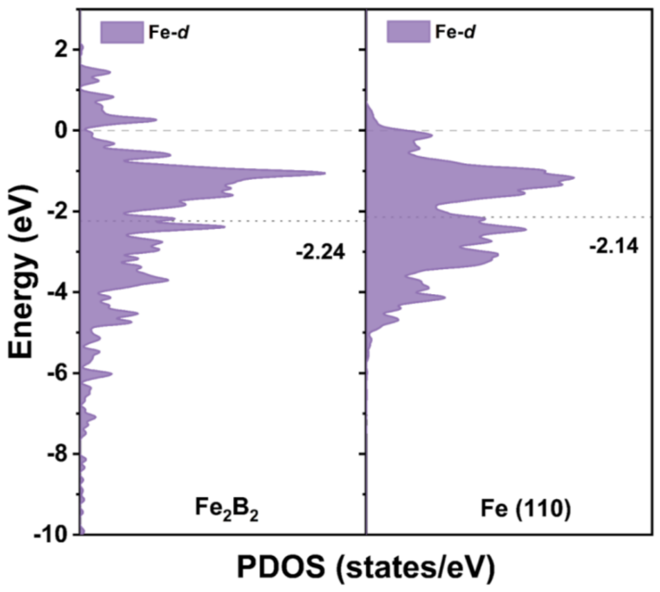

Figure S3. The partial density of states (PDOS) plots of Fe_2B_2 and Fe (110). The *d*-band centers are also highlighted in the PDOS curves.

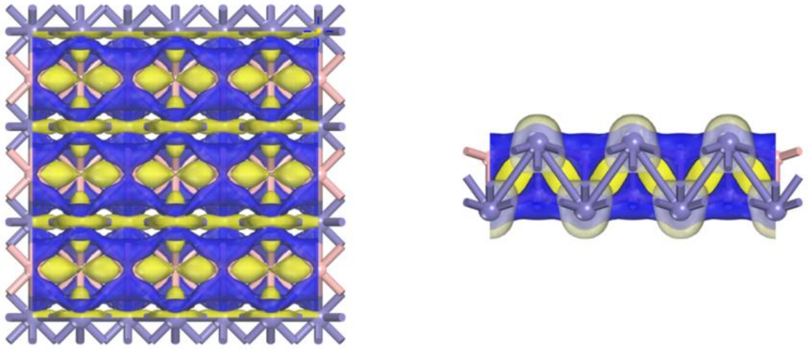

Figure S4. The atomic electron density maps of Fe_2B_2 surface. The blue and yellow isosurfaces represent electron density accumulation and depletion, respectively.

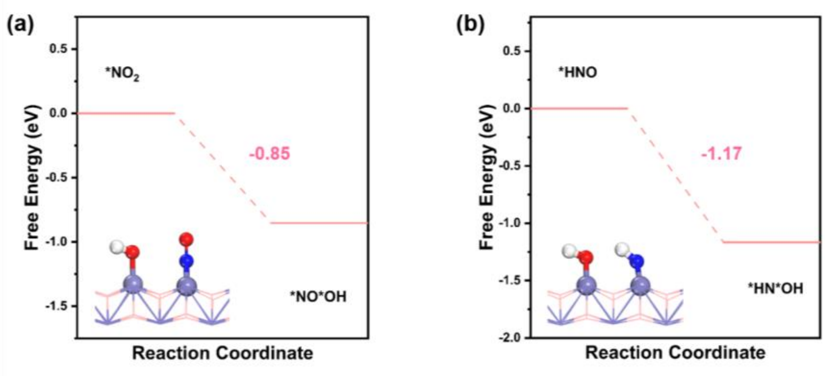

Figure S5. (a) The reaction free energy changes of $*NO_2$ to *NO*OH and the corresponding structure on Fe_2B_2 surface. (b) The reaction free energy changes of *HNO to *HN*OH and the corresponding structure on Fe_2B_2 surface.

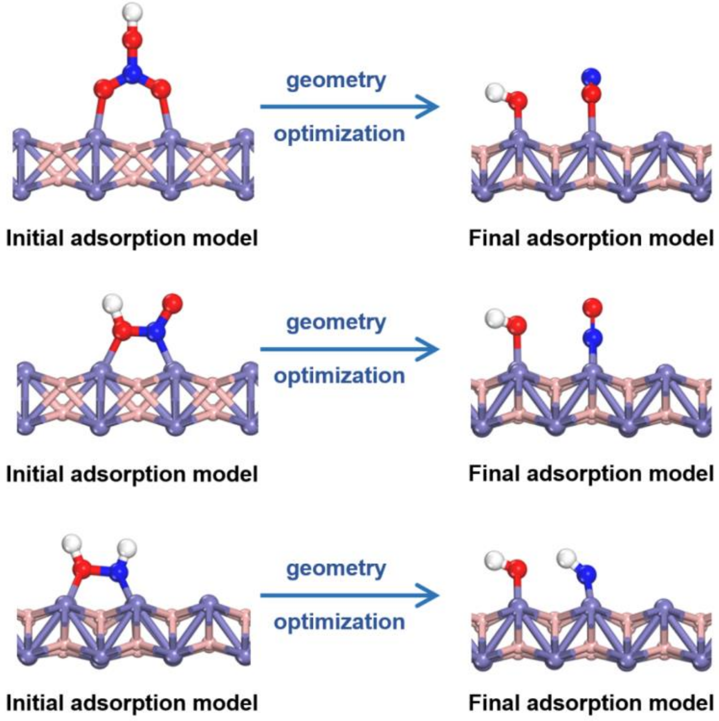

Figure S6. The corresponding adsorption configurations of $*NO_3H$ ($*NO_2*OH$), $*NO_2H$ (*NO*OH) and *HNOH (*HN*OH) before (left) and after (right) geometry optimization on Fe_2B_2 surface.

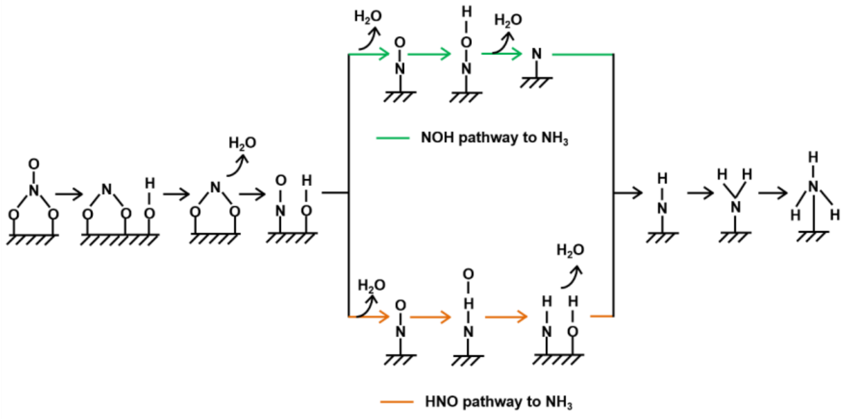

Figure S7. The brand-new reaction mechanism of intermediates separation strategy.

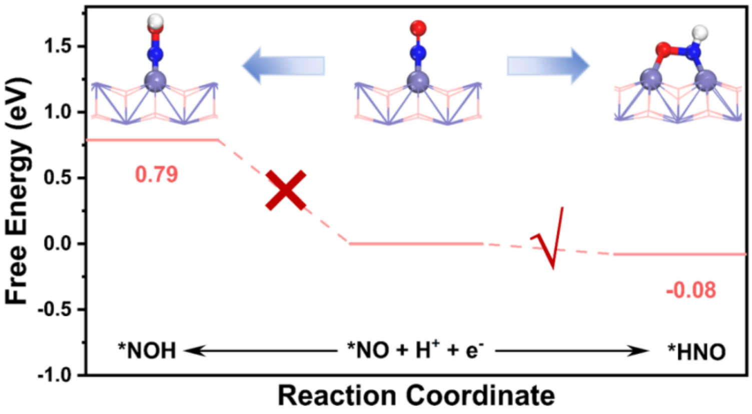

Figure S8. Protonation of *NO on Fe_2B_2, including two competitive pathways of *NOH and *HNO.

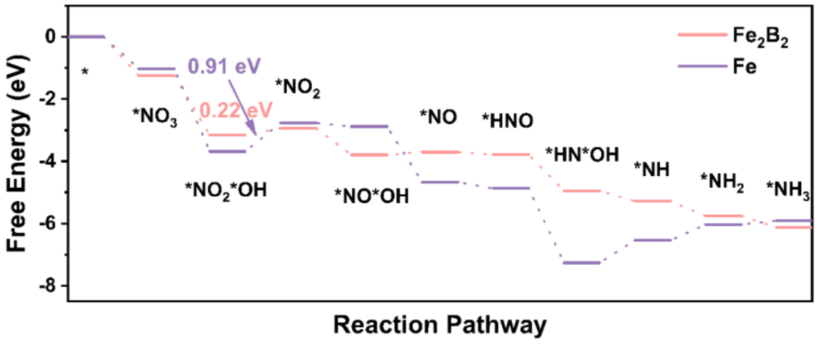

Figure S9. The Gibbs free energy change diagram for NO_3RR on Fe_2B_2 surface and Fe(110) (* represents the active site).

Commented [M1]: Please add an explanation for * in the figure.

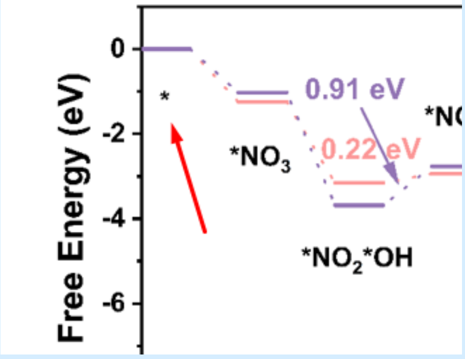

Commented [I2R1]: I have added an explanation in the highlight.

Formatted: Highlight

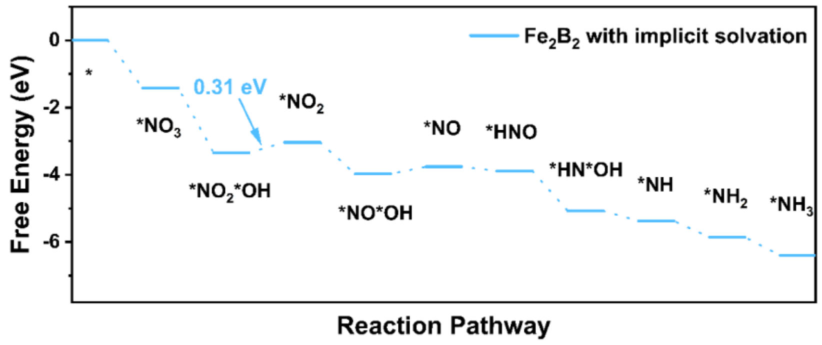

Figure S10. The Gibbs free energy change diagram for NO_3RR on Fe_2B_2 surface incorporating the implicit solvation effect (* represents the active site).

Commented [M3]: Please add an explanation for * in the figure.

Commented [I4R3]: I have added an explanation in the highlight.

Formatted: Highlight

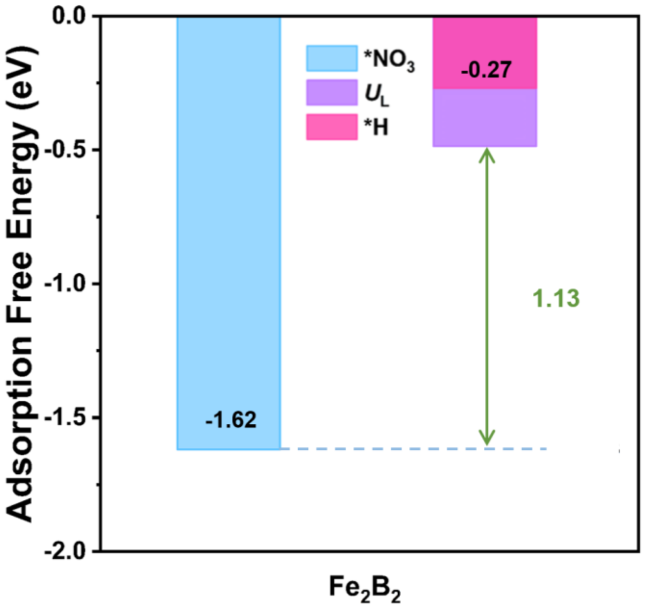

Figure S11. The calculated the adsorption energy of $*NO_3$ and *H on Fe_2B_2 surface.
